# Relationship between diversity and stability of a karst plant community

**DOI:** 10.1002/ece3.9254

**Published:** 2022-08-26

**Authors:** Yang Wang, Jin Chen, Limin Zhang, Ling Feng, Linbin Yan, Fangbing Li, Xiangwei Zhao, Lifei Yu, Na Liu

**Affiliations:** ^1^ Key laboratory of Plant Resource Conservation and Germplasm Innovation in Mountainous Region (Ministry of Education) College of Life Sciences/Institute of Agro‐Bioengineering, Guizhou University Guiyang Guizhou Province China; ^2^ Institute of Mountain Resources of Guizhou Academy of Sciences Guiyang China; ^3^ Guizhou Academy of Forestry Sciences Guiyang China

**Keywords:** functional diversity, functional redundancy, karst, recovery, stability

## Abstract

The relationships among species diversity, functional diversity, functional redundancy, and community stability are central to community and ecosystem ecology. In this paper, a “space substitution for time” approach is used to study the plant communities at different stages of the natural recovery process of degraded karst vegetation on the karst plateau of Guizhou. These restoration stages include the herbaceous stage, herbaceous and shrub transition stage, shrub stage, tree and shrub transition stage, and tree stage. We calculated the functional diversity and functional redundancy of the community based on functional characteristics and mediated the relationship between functional diversity, functional redundancy, and stability of the plant community through changes in functional diversity and functional redundancy. This study aims to reveal the mechanisms of changes in species diversity and community stability and thus further reveals the intrinsic reasons for maintaining the stability of karst plant communities. The most important results include the following: (1) Species diversity, functional redundancy, and stability gradually increased with restoration, and there were significant differences among the different stages; functional diversity increased at first and then decreased, and reached the highest level at the tree and shrub transition stage; (2) Plant height and specific leaf area were functional traits that influenced the diversity and stability of the plant community, with plant height being positively correlated with plant community diversity and stability, and specific leaf area being negatively correlated with plant community diversity and stability; (3) During the community's recovery, functional diversity and functional redundancy interacted to maintain stability. In the early and late stages of recovery, the effect of functional redundancy on stability was greater than that of functional diversity, but it was the opposite in the middle stages; (4) The tree and shrub transition stage is the likely point at which the functional diversity of plant communities in karst areas reaches saturation, and the growth rate of functional redundancy after functional diversity saturation is greater than that before saturation. Overall, community stability increased with species diversity; habitat heterogeneity increased functional diversity in the early stages of recovery; and habitat homogeneity increased functional redundancy.

## Graphical abstract

This paper examines plant communities at different stages of vegetation restoration in the Guizhou karst plateau to study the relationship among functional diversity, functional redundancy, and stability of plant communities. The most important results include the following. (1) Species diversity (SD), functional redundancy (FR), and stability (STB) gradually increased with restoration, and there were significant differences among the different stages; (2) During the community recovery, FD and FR interacted to maintain stability. In the early and late stages of recovery, the effect of functional redundancy on stability was greater than that of functional diversity, but it was the opposite in the middle stages; (3) The tree and shrub trasiti stage is the likely point at which the functional diversity of plant communities in karst areas reached saturation.

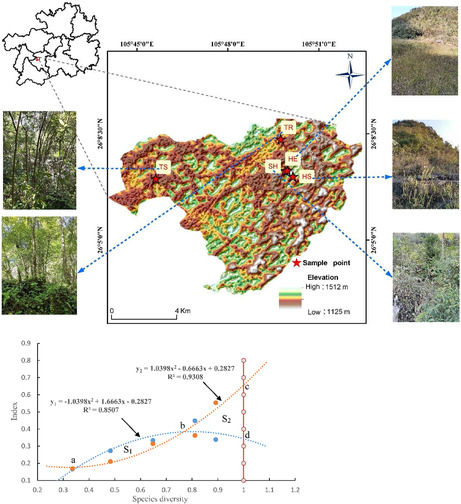

## 1 INTRODUCTION

With the acceleration of global species extinctions (Butchart et al., 2010), the correlation between species diversity and ecosystem stability has become an important scientific issue (Macarthur, 1955; Elton, 2000; Mougi & Kondon, 2012). Species diversity is an important part of biodiversity (Crawley & Harral, 2001), not only in terms of providing material, energy, and stability to ecosystems (Ives & Carpenter, 2007; Tilman, 1994), but also in terms of directly affecting the structure and function of communities (Hart et al., 2016; Levine et al., 2017).

The mechanisms underlying changes in community stability include compensation dynamics (Ives, 1995; Tilman, 1996), mean–variance scaling relationships (Doak et al., 1998; Taylor, 1961), and dominant species effects (Grman et al., 2010; Polley et al., 2007; Sasaki & Lauenroth, 2011). Compensatory dynamics mainly include the regulation of stability mechanisms by competition and the asynchrony of species responses. It has been found that competition among species can make system properties more unstable, so increasing system stability requires weakening the intensity of interspecific competition (Loreau & de Mazancourt, 2013). The asynchrony of species response is mainly reflected in two aspects—in the opposite direction of response to environmental fluctuations and in the different speeds of response after the occurrence of environmental fluctuations. Species improve the stability of the community through the change of multiplicity, the differentiation of ecological niches, and the change of biomass under specific environmental fluctuations (Hector et al., 2010). Natural communities are composed of different species that interact with each other while their multiplicity fluctuates stochastically over time. If species are assumed to be independent of each other, the sum of species covariates is zero, so some mechanisms that cause changes in the sum of variances must play a role in the relationship between biodiversity and stability (Sasaki & Lauenroth, 2011). Taylor (1961) found that there is an exponential relationship between the mean of variance and multiplicity in biomes, and that community stability increases as species diversity increases. In natural systems, the functional properties of the system are largely determined in the short term by the nature of the dominant species, and some studies, especially field experiments, have found that dominant species can limit the relationship between biodiversity and stability (Polley et al., 2007; Sasaki & Lauenroth, 2011). Some studies have shown that dominant species are more stable than other species (Grman et al., 2010; Polley et al., 2007) and that increasing the relative abundance of stable species increases the stability of communities (Grman et al., 2010). All of these mechanisms can underlie patterns of biodiversity. Interest in the relationship between diversity and stability increased in the 1950s. Based on repeated observations, Odum (1953) suggested that population density was more likely to fluctuate drastically in simple terrestrial communities than in complex communities. Elton (1958) showed that communities with high diversity are more resistant to invasion by exotic species. Gardner and Ashby (1970) and May (1973) showed that diversity reduces the stability of systems through mathematical modeling, which is in contrast to the results of previous studies. Subsequently, some ecologists failed to find a significant relationship between diversity and stability (Goodman, 1975). Thus, there is currently no unified understanding of the relationship between diversity and stability across ecosystems (Elton, 1958; Ives & Carpenter, 2007; Li et al., 2013; Maestre et al., 2012; Mccann, 2000).

One way species diversity can be quantified is based on functional diversity and functional redundancy (de Bello et al., 2007; Díaz et al., 2007), such that species diversity (SD) = functional diversity (FD) + functional redundancy (FR). Functional diversity is a key factor linking species diversity and ecosystem function (Flynn et al., 2011), and plays an important role in the relationship between environmental change, community composition, and ecosystems (Mori et al., 2013). When community species have completely different functional characteristics and each function is unique, the loss of any species can have a significant impact on community stability, which, as predicted by the riveting hypothesis, should depend entirely on functional diversity (de Bello et al., 2007). In addition, Wood et al. (2017) examined two common small mammals using stable isotope and functional trait dendrograms to determine whether FD is associated with short‐term population stability and small mammal community stability, and to test whether spatially explicit trait filters help explain the observed FD patterns. Ultimately, it is proposed that functional diversity can influence stability (Wood et al., 2017). When functional diversity shows an asymptotic pattern with the increase of species diversity, it indicates the existence of functional redundancy (Petchey et al., 2007; Pillar et al., 2013; Sasaki et al., 2009). Functional redundancy represents species in ecosystems performing similar functions (Pillar et al., 2013), where species loss would not substantially affect ecosystem function (Rosenfeld, 2002). The existence of functional redundancy can facilitate the recovery of a system following a disturbance (Walker, 1992; Walker, 1995). Therefore, when assessing the stability of plant communities, redundancy theory may be a more reliable metric than diversity (Tilman, 1994).

Quantitative functional redundancy (de Bello et al., 2007) research focuses on plant communities. For example, Ricotta et al. (2016, 2018, 2020) revealed functional redundancy of systems based on α‐diversity and β‐diversity. However, few reports focus on the stability of different recovery stages based on functional diversity and functional redundancy. Karst forests are fragile ecosystems (Yu et al., 2002), and their distribution pattern is the product of multiple ecological processes (Song et al., 2015). The Southwest Karst region is one of the most fragile ecological regions in China, and its more serious stone desertification is a constraint on the ecological environment in the region (Wang, 2003). Vegetation restoration is the fundamental way to manage karst stone desertification, and plant community stability is an important element of vegetation restoration research (Ren et al., 2007). Relevant studies have been reported on the restoration of plant ecosystems in karst areas, such as the structure of forest vegetation communities during the restoration process and the relationship between plants and the environment (Pocock et al., 2012; Yu et al., 2000; Yu et al., 2002). However, there are few studies on the stability of karst plant communities during restoration, especially the study of species diversity on the stability of karst plant communities. Here, the effects of functional diversity and functional redundancy on the stability of karst plant communities were examined by addressing the following questions: (1) What is the relationship between species diversity and community stability? (2) What is the relationship of species diversity with functional diversity and functional redundancy? (3) What role does functional diversity or functional redundancy play in maintaining community stability?

## 2 METHODS

### 2.1 General situation of the study area

**FIGURE 1:**
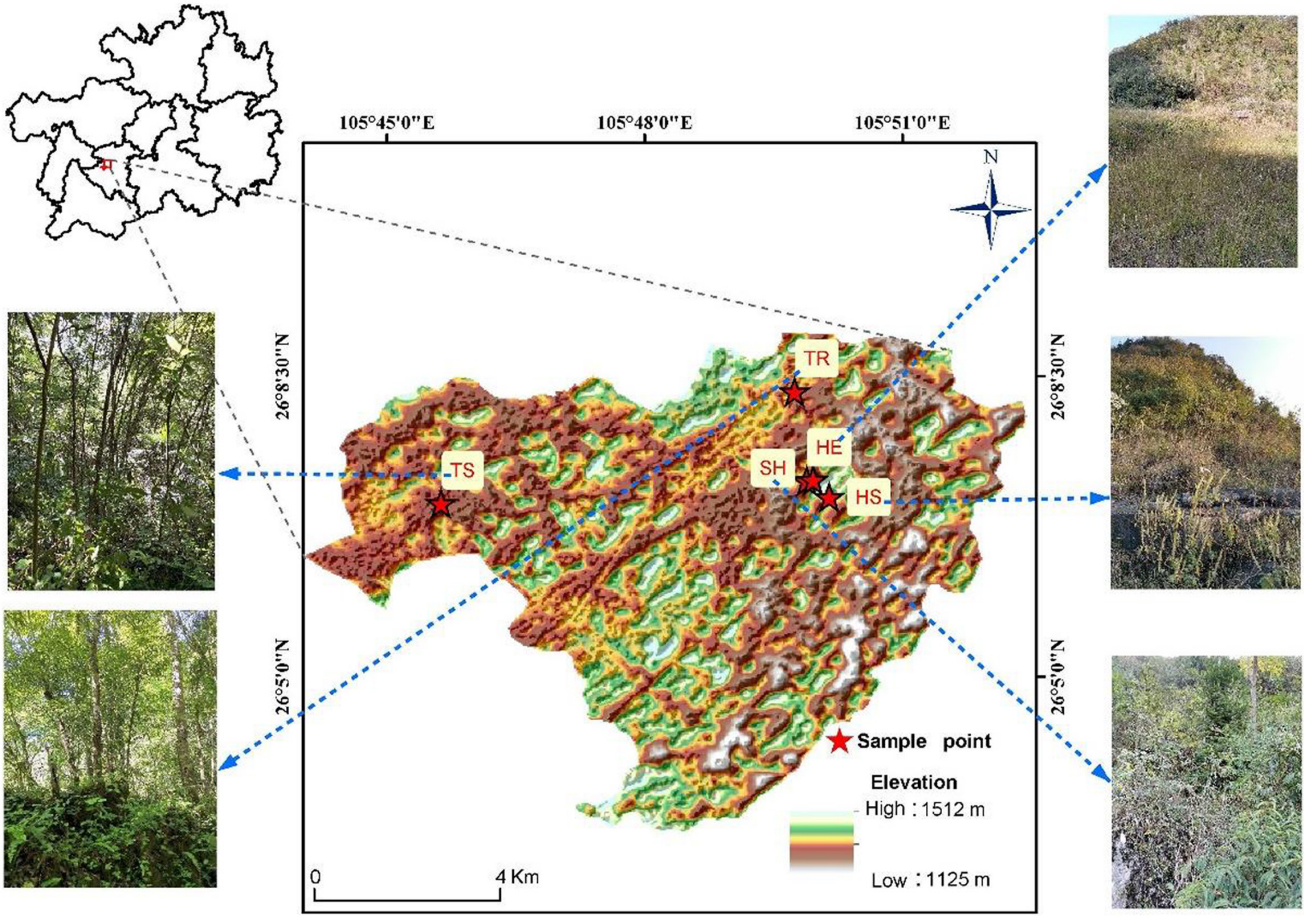
Geographic location and sampling sites of the study area. HE, herbaceous stage; HS, herbaceous and shrub transition stage; SH, shrub stage; TS, tree and shrub transition stage; TR, tree stage.

The study area is on a typical karst plateau in central Guizhou Province, China, which is located in Zhenning County, Anshun City (Figure 1). There are plant communities in different restoration stages (This is a recovery process after a disturbance) in this area (Liu et al., 2020). Zhenning County is located at 105°35′–106°01′ east longitude and 25°25′–26°11′ north latitude. Its topography is high in the north and low in the south, and the slope varies greatly (altitude of 447–2177 m). The region has a subtropical humid monsoon climate with an annual average temperature of 16.2°C, an annual sunshine duration of 1142 h, and an annual average precipitation of 1277 mm. The vegetation in the study area has undergone a gradual natural recovery process after the forests were deforested in 1958–1960. The area is a karst landscape with a consistent soil development type, that has been studied by several scholars (Liu et al., 2019; Liu et al., 2020). The parent rock of the soil formation is limestone, and the soil type is limestone. The vegetation in the study area is mainly mixed evergreen deciduous broadleaf forest and evergreen broadleaf forest. The forest vegetation in the area has been damaged to varying degrees due to human activities. After 1960, the government banned the felling of trees and promoted the natural recovery of vegetation, so that plant community types with different recovery stages exist in the area. Due to the anthropogenic disturbance in different years of this process, the vegetation communities can be classified into herbaceous stage (HE), herbaceous and shrub transition stage (HS), shrub stage (SH), tree and shrub transition stage (TS) and tree stage (TR) according to the appearance of the vegetation. We chose these five different recovery stages as sampling points to better analyze the relationship between species diversity and stability under each stage and help understand the characteristics of changes from the previous to the next recovery stage. The dominant species in HE were *Imperata cylindrica*, *Carex capilliformis*, and *Miscanthus sinensis*; the dominant species in SH were *Pittosporum tobira*, *Pyracantha fortuneana*, *Rosa cymosa*, and *Coriaria nepalensis*; and the dominant species in TR were *Platycarya strobilacea*, *Carpinus pubescens*, *Cyclobalanopsis argyroscopia*, and *Celtis sinensis*.

### 2.2 Plot set‐up and community investigation

The sampling of plots was completed from May to June in 2019 and from October to November in 2020 using a “space substitution for time” approach (Mueller‐Dombois & Ellenberg, 1974). In the study area, we selected sample sites with similar conditions, such as elevation, slope, and aspect. We determined the sampling area for each recovery stage based on the “species‐area curve” (Feng et al., 2011). It is usually used as a reference to determine the size of the survey sample. Affected by the geological environment, the soil in karst areas is thin and vulnerable to soil erosion or soil loss. Therefore, the vegetation growth in karst areas has discontinuous characteristics, which is different from the vegetation growth in other areas. Finally, we obtained a minimum sampling area of 400 m^2^ for the TR and TS stages, 100 m^2^ for the SH and HS stages, and 25 m^2^ for the HE stage. In the TR and TS stages, a 20 m × 20 m sample plot was established; in the SH and HS stages, a 10 m × 10 m sample plot was established; in the HE stage, a 5 m × 5 m sample plot was established; there were three replicate sample plots in each stage for a total of 15 sample plots. In general, the distance between three replicate sites needs to be >100 m, but the interval should not be too large, and try to have the same elevation, slope, and direction (Liu, Yu, Zhao, Wu, & Yan, 2020; Liu, Yu, Yan, Liu, & Zhao, 2019).

In the sampling method in this paper, we refer to the nested sampling method which is suitable for karst forest community proposed by Yu et al. (2000). Specifically, in each restoration stage, all the tree layers were investigated first, then the shrub layers in the plot area were investigated, and finally the herb layers in the plot area were investigated. The area of the four tree sample plots was 10 m × 10 m, the area of the four shrub sample plots was 4 m × 4 m, and the area of the four herb sample plots was 1 m × 1 m. A total of 24 tree layer samples, 24 shrub layer samples, and 24 herbaceous layer samples were investigated for the TS and TR stages; 24 shrub layer samples and 24 herbaceous layer samples were investigated for SH and HS stages; and 12 herbaceous layer samples were investigated for the HE stage. A total of 132 samples, 24 tree layer samples, 48 shrub layer samples, and 60 herbaceous layer samples, were investigated for all five restoration stages. The latitude and longitude of each sample were recorded. We recorded the identity and height of all plants, the diameter at breast height and crown width of trees and shrubs, and the ground diameter and number of herbs. We photographed and recorded the characteristic traits of the species such as leaves, flowers, and fruits, and then compared the botanical histories and the botanical histories of botany in Guizhou to find out the names of the species. The breast diameter of a plant is measured by measuring its chest circumference with a tape measure about 1.3 m above the ground. The method of measuring the ground diameter is to measure the ground diameter of the plant with a tape measure about 0.1 m from the ground. The width of the crown is measured by measuring the shaded parts of the plant's leaves and branches that are cast vertically on the ground. Coverage is estimated as a percentage of the distribution area of herbaceous plants in the sample plot. In total, we surveyed and recorded 146 species.

### 2.3 Selection and determination of functional traits

Six quantitative traits were chosen, including plant height (PLH), leaf thickness (LT), chlorophyll content (CHL), leaf dry matter content (LDMC), leaf area (LA), and specific leaf area (SLA). These were assessed following the Manual of Standardized Measurement of Global Plant Functional Traits (Pérezharguindeguy et al., 2013). For trees under 5 m, we can directly measure with a tape measure. For trees above 5 m, a pole of known length is set up on the ground according to the sun's irradiation, and the shadow length of the tree and the shadow length of the pole are measured separately, and then obtained according to the proportional relationship (the height of the tree is equal to the height of the pole multiplied by the shadow length of the tree and then divided by the shadow length of the pole). Species showing healthy growth in each sample were identified, and 10 healthy leaves of each species (from different locations on the plant) were collected using shears and placed in self‐sealing bags for measurements of functional traits. LT was measured using electronic vernier calipers (Derek, DL91150). Leaf length and leaf area (LA) were calculated using a scanner and Photoshop software (HP, HPScanJetN92120), and the chlorophyll content (CHL) of leaves was determined using a chlorophyll meter (Linde, LD‐YD). For leaves with a near‐cylindrical shape, the diameter and length were determined using digital vernier calipers, and the total leaf area was calculated using the formula for calculating the surface area of a cylinder. Ten healthy leaves of each species were selected, the petioles were removed, and the fresh weight (FW) of these leaves was weighed; these leaves were then dried in an oven at 60°C for 72 h. The dry weight (DW) of these leaves was then weighed, and the leaf dry matter mass was equal to the ratio of leaf dry weight to leaf fresh weight (i.e., LDMC = DW/FW). The ratio of leaf area to dry weight is equal to the specific leaf area (SLA) (i.e. SLA = LA/DW).

### 2.4 Data calculation and statistical analysis

#### 2.4.1 Functional redundancy calculation

We use the algorithm proposed by de Bello et al. (2007) to calculate the size of functional redundancy.

$$\mathrm{SD}=1-\sum_{i=1}^{S}\frac{N_{i}\left(N_{i}-1\right)}{N\left(N-1\right)}$$

$$\mathrm{FD}=\sum_{i=1}^{S}\sum_{j=1}^{S}d_{\mathrm{ij}}p_{i}p_{j}$$

FR=SD−FD

In the formula, SD is the Simpson diversity index (Simpson, 1949), FD is Rao's coefficient (Rao, 1982), and *S* is the number of species in the sample. *N* represents the total number of individuals of all species and *N*_*i*_ represents the number of individuals of the *i*th species; *p*_*i*_ and *p*_*j*_ are the relative densities of the *i* and *j* species in the sample; and *d*_*ij*_ is the Euclidean distance, 0 ≤ *d*_*ij*_ ≤1, indicating the difference of species *i* and *j* in a set of character spaces (Gower, 1971).

#### 2.4.2 Calculation of community stability

Community stability is expressed by the reciprocal *STB* of the variation of species population density:

$$\mathrm{STB}=\frac{\mu }{\sigma }$$

In the formula, *μ* is the average density of each species in the sample and *σ* is the standard deviation of the density of each species. The higher the STB value, the higher the community stability, because the variability of each species density was smaller than the average density variability among species (Lehman & Tilman, 2000). The improved Godron stability method was used to determine community stability (Lv et al., 2007).

#### 2.4.3 Statistical analysis

We used SPSS 22.0 software for statistical analysis. Since the sample data satisfy the following conditions: the samples are independent of one another and their overall variances when compared to one another are equal. Therefore, one‐way anova was used for changes in diversity and stability at different recovery stages (We use the check method of Shapiro–Wilk to test the normality distribution of the data, and only when the data provide the test of normal distribution can we carry out one‐way anova), multiple comparison tests were performed, and ggpplot2 was used to visualize the data (Wickham, 2016).

A Pearson correlation analysis was used to determine the correlation between plant communities in the study area and functional traits, diversity as well as stability under different recovery stages, and the output was visualized in Figure 3 after graphing with R4.0.3 (R Core Team, 2020). The R package used is “corrplot” and “cor.mtest” (Wei & Viliam, 2021).

Binary stepwise regression analysis was used to determine the relative influence of functional diversity and functional redundancy on stability, using community stability as the dependent variable and functional diversity and functional redundancy as independent variables. The degree of influence was determined by standardized partial regression coefficients. This analysis was used to analyze the magnitude of community stability affected by functional diversity and functional redundancy at different restoration stages, which ultimately led to Table 1.

**TABLE 1:** Stepwise regression analysis of the effects of functional diversity (x1) and functional redundancy (x2) on community stability (y) at different restoration stages.

Recovery stages	Regression equations	*R* ^2^	*p*
HE	y = 0.048 + 1.003x1 + 1.058x2	.776	.011
HS	y = 0.067 + 0.869x1 + 0.834x2	.938	<.001
SH	y = 0.013 + 0.934x1 + 0.800x2	.969	<.001
TS	y = 0.026 + 0.982x1 + 0.779x2	.732	.019
TR	y = 0.890 + 0.284x1 + 0.415x2	.909	.001

*Note*: *R* ^2^ represents the goodness of fit of the equation, the closer the fit is to 1, the better the equation fits. When P represents the regression equation's significance for the *F* test, *p* < .05 indicates that the regression model is reliable and the data are ecologically significant. The abbreviations of the recovery stages are shown in Figure 1.

The relationship between functional diversity and species diversity, functional redundancy and species diversity was analyzed by using scatter plot in regression analysis. Assuming that the species diversity can reach the maximum value 1, after fitting the species diversity, functional diversity, and functional redundancy functions in Origin 9.1 software, the “screen reader” is used to read the intersection coordinates to obtain the intersection coordinates a, b. The values of intersection coordinates c and d are obtained by mathematical calculation. In MatLab 7.0, the “solve” function is used to calculate the area of the two fitted curves, and the area S_1_ is obtained, and the area of any closed curve is calculated by “PolyArea” based on b, c, and d coordinates, and the area S_2_ is obtained (Figure 4).

## 3 RESULTS

### 3.1 Characteristics of diversity and stability in different restoration stages

**FIGURE 2:**
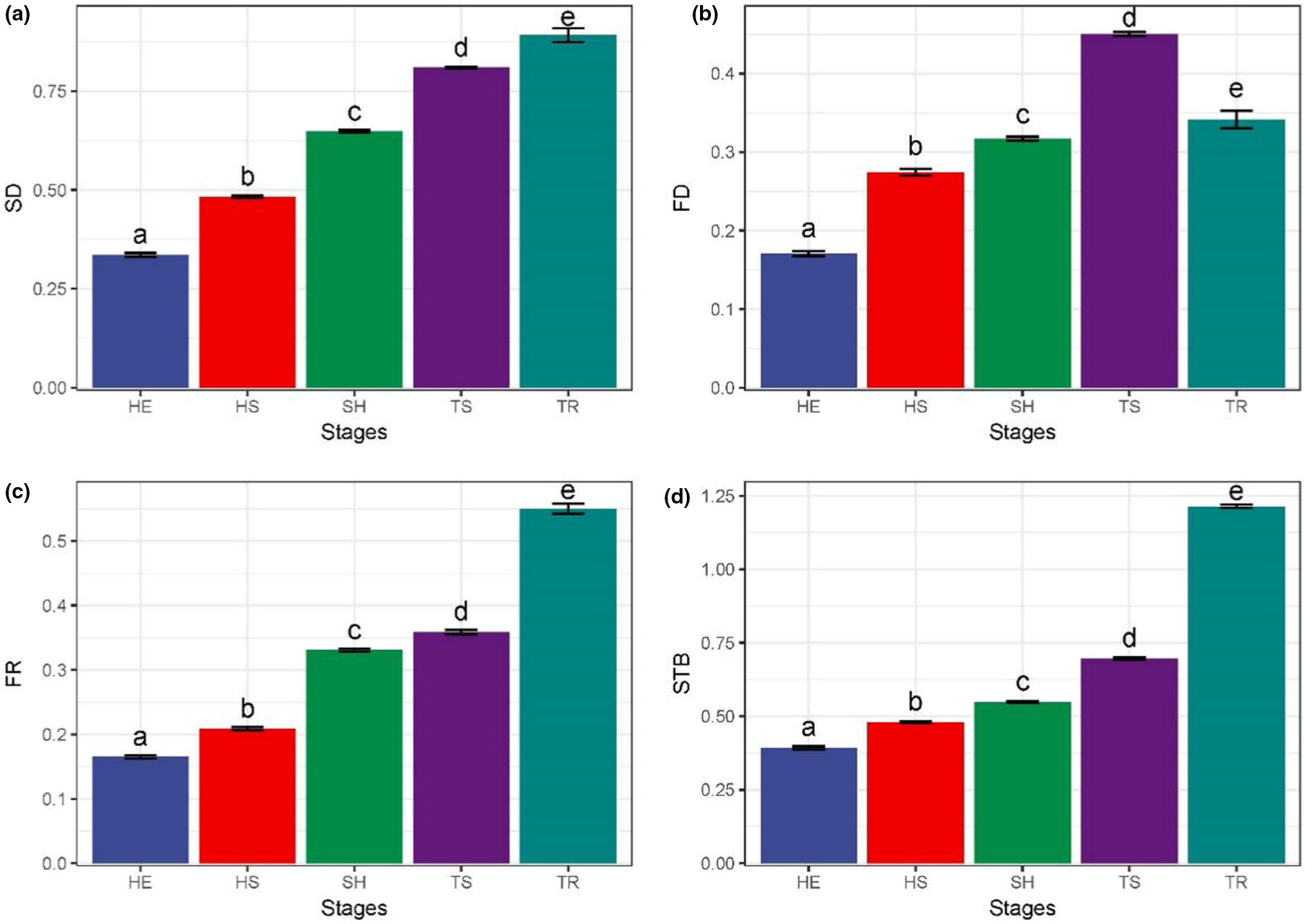
Characteristics of changes in species diversity, functional diversity, functional redundancy, and stability at different stages of recovery. The vertical coordinate represents the index and the horizontal coordinate represents the different recovery stages. Bar graphs show the mean (±95% confidence interval—CI); the error bar is the standard error (SD). Three replicates were performed for each recovery stage. (a) SD, species diversity; (b) FD, functional diversity; (c) FR, functional redundancy; (d) STB, stability. Multiple comparison tests were used to determine statistical significance, which indicates statistical significance when *p* < .05. Different lowercase letters between (a–e) represent significant differences. The abbreviations of the recovery stages are shown in Figure 1.

The trends of SD, FR, and STB were consistent with the progress of plant community restoration (*p* < .001). They showed significant differences at different restoration stages and gradually increased with the progress of restoration. As shown in Figure 2, SD, FR, and STB were lowest in the herbaceous stage and highest in the tree stage. The SD, FR, and STB for the HE stage were 0.34, 0.16, and 0.39, while the SD, FR, and STB for the TR stage were 0.89, 0.55, and 1.21. The FD increased and then decreased as the restoration progressed, reaching a maximum value of 0.45 (*p* < .001) in the arboreal and shrub transition stage.

### 3.2 Correlation of functional traits with diversity and stability

**FIGURE 3:**
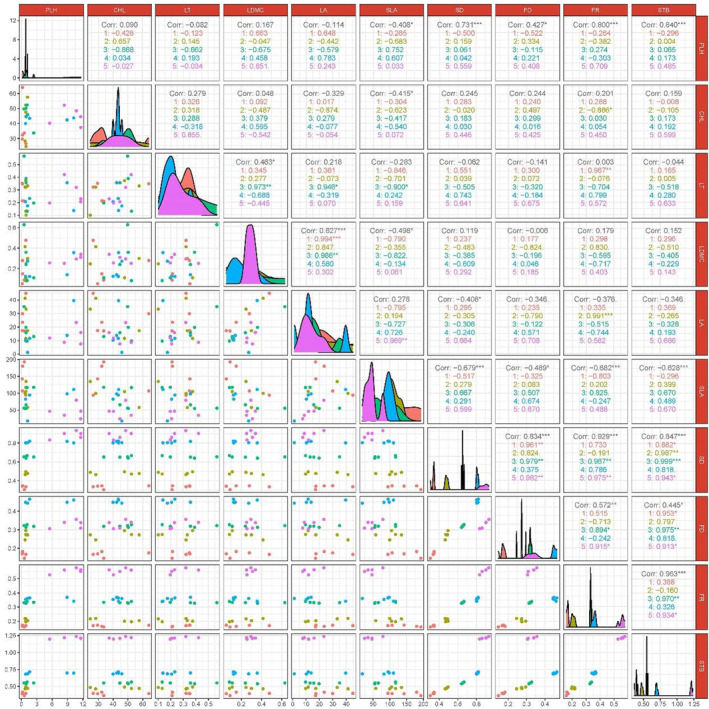
Pearson correlation diagram of functional traits with diversity and stability. ***p < .001; **p < .01; *p < .05. Corr represents the correlation coefficients between functional traits, SD, FD, FR, and STB in the whole plant community. 1‐herbaceous stage, 2‐herbaceous and shrub transition stage, 3‐shrub stage, 4‐tree and shrub transition stage, 5‐tree stage. The numbers after 1, 2, 3, 4, 5 represent the correlation coefficients of the corresponding stages. Figure 2 depicts the abbreviations SD, FD, FR, and STB. PLH, plant height; LT, leaf thickness; CHL, chlorophyll content; LDMC, leaf dry matter content; LA, leaf area; SLA, specific leaf area.

In terms of the overall plant community, SD was negatively correlated with LA (*p* < .05) and SLA (*p* < .001), SD was significantly positively correlated with PLH (*p* < .001), and STB (*p* < .001) was significantly negatively correlated with SLA (*p* < .001). FD was significantly negatively correlated with SLA (*p* < .05) and positively correlated with PLH (*p* < .05). FR was highly negatively correlated with SLA (*p* < .001) and significantly positively correlated with PLH (*p* < .001). STB was significantly positively correlated with FD (*p* < .05), FR (*p* < .001), and SD (*p* < .001), and the degree of correlation indicated that FR(0.96) > SD(0.85) > FD(0.45). According to the display in Figure 3, in the SH and TR stages: SD was significantly and positively correlated with FD (*p* < .01), FR (*p* < .001), and STB (*p* < .001); FD was significantly and positively correlated with FR (*p* < .01) and STB (*p* < .05); and FR was significantly and positively correlated with STB (*p* < .001).

### 3.3 Functional diversity, functional redundancy, and stability relationships in different recovery phases

Refer to Table 1, In the HE stage, the biased regression coefficient (x2) of functional redundancy was 1.058, and the biased regression coefficient (x1) of functional diversity was 1.003; the biased regression coefficient of functional redundancy in this stage was greater than that of functional diversity. In the HS stage, SH stage, and TS stage, the biased regression coefficients (x2) of functional redundancy were 0.834, 0.800, and 0.779. The biased regression coefficients (x1) of functional diversity were 0.869, 0.934, and 0.982, and the biased regression coefficients of functional redundancy in these three stages were smaller than those of functional diversity. In the TR stage, the biased regression coefficient (x2) of functional redundancy was 0.415 and the biased regression coefficient (x1) of functional diversity was 0.284, and the biased regression coefficient of functional redundancy in this stage was greater than that of functional diversity.

### 3.4 Characteristics of functional diversity and functional redundancy with species diversity

**FIGURE 4:**
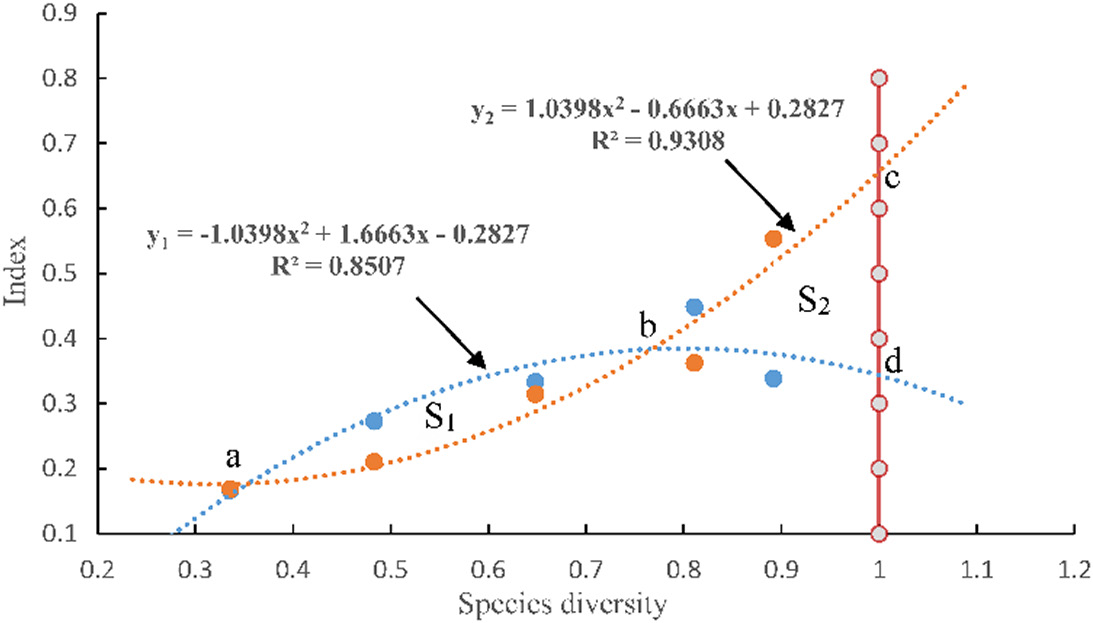
Curves of functional diversity and functional redundancy with species diversity. The blue dashed line is the change in functional diversity with species diversity (y_1_); the orange dashed line is the change in functional redundancy with species diversity (y_2_); the red line is when the species diversity index is 1. *R* ^2^ represents the interpretation rate of the fitted curve. “a” represents the first equal value of functional diversity and functional redundancy as species diversity increases; “b” represents the second equal value of functional diversity and functional redundancy as species diversity increases; “c” represents the value of functional redundancy when species diversity is at its maximum; and “d” represents the value of functional diversity when species diversity is at its maximum. S_1_ represents the amount of increase in functional diversity before functional diversity saturation, and S_2_ represents the increase in functional redundancy with functional diversity saturation.

As can be seen from Figure 4, the explanation rate of the two fitted curves is at least 0.8507, which indicates that the two fitted curves have a good fitting effect. These two fitted curves indicated that functional diversity showed an increasing and then decreasing change with increasing species diversity, while functional redundancy showed a gradual increasing trend with increasing species diversity.

In addition, the coordinates of the intersection points in the graphs are indicated as a (0.35, 0.18), b (0.77, 0.38), c (1, 0.66), d (1, 0.34);. S_1_ = 0.0244, S_2_ = 0.0364.

- When SD < 0.35, y_2_ > y_1_, indicating that the increase in functional redundancy is faster than the increase in functional diversity.
- When 0.35 < SD < 0.77, y_2_ < y_1_, indicating that the increase of functional diversity is faster than the increase of functional redundancy.
- When 0.77 < SD, y_2_ > y_1_, indicating that the increase in functional redundancy is faster than the increase in functional diversity.

This shows that FD reaches its maximum at point b with a value of 0.38 and FR reaches its maximum at point c with a value of 0.66. Therefore, we define point b as the saturation point of functional diversity. After the saturation of functional diversity, functional redundancy will exceed functional diversity as species diversity increases.

## 4 DISCUSSION

### 4.1 Effect of species diversity on stability at different restoration stages

Numerous studies have shown that species diversity has a positive effect on community stability (Cottingham et al., 2001; Isbell et al., 2009; Tilman, 1999; Tilman et al., 2006; Valone & Hoffman, 2003). The species diversity of plant communities in this study (Figure 2) increased with the process of restoration (This is a recovery process after a perceived disturbance), which is consistent with the findings of Yu et al. (1998, 2002). It has been shown that as restoration proceeds, the ecology becomes more favorable for the settlement of other species (Sitzia et al., 2015). As the community becomes richer in species and more complex in structure, its stability may increase (Campbell et al., 2011; Naeem & Li, 1997). The present study (Figure 3) showed a highly significant positive correlation between SD and STB, indicating that species diversity and stability of degraded karst plant communities are closely related during the restoration process.

### 4.2 Impact of functional diversity and functional redundancy on stability in different recovery stages

Species diversity is the basis for generating functional diversity and functional redundancy, with functional diversity acting as a complementary effect and functional redundancy as an insurance effect (Tilman, 1999). Plant functional traits can help identify the adaptive responses and resource allocation strategies of plants (Guittar et al., 2016); therefore, exploring functional traits, functional diversity, and functional redundancy can elucidate system stability, community structuring mechanisms, and community productivity (He et al., 2018). It has been shown that plants generally exhibit low specific leaf area and high leaf dry matter content to adapt to habitats, such as shallow karst soils with high soil water seepage (Biswas et al., 2019; Chen et al., 2016; Dunck et al., 2016; Niu et al., 2014). Lower specific leaf area indicates higher plant access and utilization of resources, such as light, water and is closely related to plant survival strategies (Ali et al., 2017; Garnier et al., 2001; Gazol & Camarero, 2016). The results of this study showed (Figures 2 and 3) that PLH was significantly and positively correlated with FD, FR, and STB, and SLA was significantly and negatively correlated with FD, FR, and STB. This indicates that as the recovery proceeds, the plant community's access to resources and photosynthetic capture capacity increases, promoting the increase of FD, FR, and STB.

Pillar et al. (2013) found that community stability was maintained only by functional redundancy, while functional diversity had no effect on stability in a study of grazed grasslands in southern Brazil. Our study shows that both functional redundancy and functional diversity exist simultaneously during plant community restoration and maintain stability together, but they have different effects on stability. However, the effect of functional redundancy on community stability was more important after the plant community entered the TS stage, which is consistent with Pillar's results (Pillar et al., 2013). Sasaki and Lauenroth (2011) found that the maintenance of community stability by functional diversity was stronger than that by functional redundancy in a study of grasslands in Mongolian pastures, and this finding is similar to some of our findings. The recovery process of plant communities from HE to TS reflects the greater importance of functional diversity for the maintenance of stability.

During the restoration of plant communities in karst areas, the habitat gradually changes from being heterogeneous to being homogenous (Yu et al., 2000). Combining these findings, together with our results, suggests that species diversity increases community stability; The intrinsic mechanism between species diversity and stability may be explained by habitat heterogeneity and habitat homogeneity. Habitat heterogeneity favors the increase of functional diversity, and habitat homogeneity favors the increase of functional redundancy. With the restoration of karst vegetation, habitat heterogeneity decreases and habitat homogeneity increases; It leads to a decrease in functional diversity and an increase in functional redundancy.

### 4.3 Relationship between functional diversity and functional redundancy with species diversity

During the HE stage, species diversity is relatively low (Figure 2), plants recover faster (faster growth), and most species mostly maintain stability mainly with functional redundancy because plant growth is the main function. This is consistent with the results of Pillar et al. (2013). Species diversity gradually increases in the middle stages of recovery (HS, SH, TS) (Figure 2), and the complementary effect of functional diversity increases and becomes the main factor in maintaining stability. In other words, the complementary effect among species was greater than the insurance effect, which is consistent with the findings of Yao et al. (2016) and Wang et al. (2017). At the end of recovery (TR), species diversity continued to increase, but functional diversity decreased (Figure 2). In the TR stage, although species diversity increases, the functions supported (or functional overlap) between species decreases, resulting in more redundant functional responses (Dalerum et al., 2012).

It is generally accepted that functional redundancy can be predicted when functional diversity shows a saturation increase pattern with increasing species diversity, but this description does not quantify the degree of functional redundancy in different communities (Petchey et al., 2007; Pillar et al., 2013; Sasaki et al., 2009). Our study shows (Figure 4) that the TS stage is the stage where functional diversity appears to increase in saturation, and it is predicted that from this stage onwards, the increase in functional redundancy should exceed functional diversity. To prove this idea, we assumed that species diversity approaches 1 as recovery proceeds; when species diversity reaches 1, stability is 1.26, indicating a gradual increase in stability as species diversity increases. S_1_ represents a net increase in functional diversity when 0.35 < SD < 0.77 and y_1_ > y_2_, and S_2_ represents a net increase in functional redundancy when 0.77 < SD < 1 and y_1_ < y_2_ (as shown in Figure 4). In addition, S_2_ (0.0364) > S_1_ (0.0244) indicates that functional diversity first increases and then decreases, and functional redundancy gradually increases during the recovery process of karst plant communities; after functional diversity reaches saturation, the growth rate of functional redundancy increases significantly, and functional redundancy plays a greater role than functional diversity in maintaining community stability.

## 5 CONCLUSION

In this study, the causes of community stability and the relationships of species diversity with functional diversity and functional redundancy were examined. The major conclusions are summarized below:

1. During the natural recovery of degraded karst plant communities, community stability increases with species diversity; plant community stability is maintained by a combination of functional diversity and functional redundancy.
2. In degraded karst plant communities, the TS stage might be key, given that this is the stage during which functional diversity reaches its saturation point; as recovery progresses, the role of functional redundancy in maintaining stability increases relative to that of functional diversity.
3. Functional diversity and functional redundancy jointly contribute to the maintenance of community stability via habitat heterogeneity, which increases functional diversity, and habitat homogeneity, which increases functional redundancy.

## AUTHOR CONTRIBUTIONS

**Yang Wang:** Conceptualization (lead); data curation (lead); formal analysis (lead); investigation (lead); methodology (supporting); project administration (equal); validation (lead); visualization (lead); writing – original draft (lead); writing – review and editing (lead). **Jin Chen:** Data curation (lead); formal analysis (lead). **Limin Zhang:** Data curation (lead); formal analysis (lead); methodology (equal). **Ling Feng:** Data curation (supporting); formal analysis (supporting); investigation (lead); methodology (supporting). **Lingbin Yan:** Conceptualization (equal); investigation (lead); methodology (lead); project administration (lead). **Fangbing Li:** Investigation (lead). **Xiangwei Zhao:** Investigation (supporting). **Lifei Yu:** Conceptualization (equal); funding acquisition (equal); methodology (equal); project administration (equal); resources (equal). **Na Liu:** Investigation (lead); methodology (lead).

## Funding information

This research was funded by the Construction Program of Biology First‐class Discipline in Guizhou (GNYL [2017]009), and the Project of National Key Research and Development Program of China (2016YFC0502604), and the Project of Promoted Innovation for Colleges and Universities of Guizhou Province (Qian Jiao He Collaborative Innovation [2014]01).

## CONFLICT OF INTEREST

The authors state that they have no conflicting interests.

## Data Availability

All data are openly available in the public data repository Dryad: https://doi.org/10.5061/dryad.k3j9kd58x.

## References

[ece39254-bib-0001] Ali, A. , Yan, E. R. , Chang, S. X. , Cheng, J. Y. , & Liu, X. Y. (2017). Community‐weighted mean of leaf traits and divergence of wood traits predict aboveground biomass in secondary subtropical forests. Science of the Total Environment, 574, 654–662. 27657991 10.1016/j.scitotenv.2016.09.022

[ece39254-bib-0002] Biswas, S. R. , Mallik, A. U. , Brauthwaite, N. T. , & Biswas, P. L. (2019). Effects of disturbance type and microhabitat on species and functional diversity relationship in stream‐bank plant communities. Forest Ecology and Management, 432, 812–822.

[ece39254-bib-0003] Butchart, S. H. , Walpole, M. , Collen, B. , van Strien, A. , Scharlemann, J. P. , Almond, R. E. , Baillie, J. E. , Bomhard, B. , Brown, C. , Bruno, J. , Carpenter, K. E. , Carr, G. M. , Chanson, J. , Chenery, A. M. , Csirke, J. , Davidson, N. C. , Dentener, F. , Foster, M. , Galli, A. , … Watson, R. (2010). Global biodiversity: Indicators of recent declines. Science, 328, 1164–1168. 20430971 10.1126/science.1187512

[ece39254-bib-0004] Campbell, V. , Murphy, G. , & Romanuk, T. N. (2011). Experimental design and the outcome and interpretation of diversity ‐stability relations. Oikos, 120, 399–408.

[ece39254-bib-0005] Chen, D. M. , Chen, J. H. , Chu, P. F. , Mi, J. , Hu, S. J. , Xie, Y. C. , Tuvshintogtokh, I. , & Bai, Y. F. (2016). Effect of diversity on biomass across grasslands on the Mongolian Plateau: Contrasting effects between plants and soil nematodes. Journal of Biogeography, 43, 955–966.

[ece39254-bib-0006] Cottingham, K. L. , Brown, B. L. , & Lennon, J. T. (2001). Biodiversity may regulate the temporal variability of ecological systems. Ecology Letters, 4, 72–85.

[ece39254-bib-0007] Crawley, M. J. , & Harral, J. E. (2001). Scale dependence in plant biodiversity. Science, 291, 864–868. 11157164 10.1126/science.291.5505.864

[ece39254-bib-0008] Dalerum, F. , Cameron, E. Z. , Kunkle, K. , & Michael, J. (2012). Interactive effects of species richness and species traits on functional diversity and redundancy. Theoretical Ecology, 5, 129–139.

[ece39254-bib-0009] de Bello, F. , Lepš, J. , Lavorel, S. , & Moretti, M. (2007). Importance of species abundance for assessment of trait composition: An example based on pollinator communities. Community Ecology, 8, 163–170.

[ece39254-bib-0010] Díaz, S. , Lavorel, S. , de Bello, F. , Quétier, F. , Grigulis, K. , & Robson, T. M. (2007). Incorporating plant functional diversity effects in ecosystem service assessments. Proceedings of the National Academy of Sciences of the United States of America, 104, 20684–20689. 18093933 10.1073/pnas.0704716104 PMC2410063

[ece39254-bib-0011] Doak, D. F. , Bigge, D. , Harding, E. K. , Marvier, M. A. , O'Malley, R. E. , & Thomson, D. (1998). The statistical inevitability of stability‐diversity relationships in community ecology. American Naturalist, 151, 264–276. 10.1086/286117 18811357

[ece39254-bib-0012] Dunck, B. , Algate, V. M. , Cianciaruso, M. V. , & Rodrigues, L. (2016). Functional diversity and trait–environment relationships of periphytic algae in subtropical floodplain lakes. Ecological Indicators, 67, 257–266.

[ece39254-bib-0013] Elton, C. S. (1958). The ecology of invasions by animals and plants. Journal of Range Management, 47, 1601.

[ece39254-bib-0014] Elton, C. S. (2000). The ecology of invasions by animals and plants. The University of Chicago Press.

[ece39254-bib-0015] Feng, J. C. , Shi, S. , Zhao, C. J. , & Liu, Y. (2011). Ecological Experiment. Minzu University of China Press.

[ece39254-bib-0016] Flynn, D. F. B. , Mirotchnick, N. , Jain, M. , Palmer, M. I. , & Naeem, S. (2011). Functional and phylogenetic diversity as predictors of biodiversity‐ecosystem‐function relationships. Ecology, 92, 1573–1581. 21905424 10.1890/10-1245.1

[ece39254-bib-0017] Gardner, M. R. , & Ashby, W. R. (1970). Connectance of large dynamic (cybernetic) systems: Critical values for stability. Nature, 228, 784. 5472974 10.1038/228784a0

[ece39254-bib-0018] Garnier, E. , Laurent, G. , Bellmann, A. , Debain, S. , Berthelier, P. , Ducout, B. , Roumet, C. , & Navas, M. L. (2001). Consistency of species ranking based on functional leaf traits. The New Phytologist, 152, 69–83. 35974476 10.1046/j.0028-646x.2001.00239.x

[ece39254-bib-0019] Gazol, A. , & Camarero, J. J. (2016). Functional diversity enhances silver fir growth resilience to an extreme drought. Journal of Ecology, 104, 1063–1075.

[ece39254-bib-0020] Goodman, D. (1975). The theory of diversity‐stability relationships in ecology. The Quarterly Review of Biology, 50, 237–266.

[ece39254-bib-0021] Gower, J. C. (1971). A general coefficient of similarity and some of its properties. Biometrics, 27, 857–871.

[ece39254-bib-0022] Grman, E. , Lau, J. A. , Schoolmaster, D. J. , & Gross, K. L. (2010). Mechanisms contributing to stability in ecosystem function depend on the environmental context. Ecology Letters, 13, 1400–1410. 20849440 10.1111/j.1461-0248.2010.01533.x

[ece39254-bib-0023] Guittar, J. , Goldberg, D. , Klanderud, K. , Telford, R. J. , & Vandvik, V. (2016). Can trait patterns along gradients predict plant community responses to climate change? Ecology, 97, 2791–2801. 27859101 10.1002/ecy.1500

[ece39254-bib-0024] Hart, S. P. , Schreiber, S. J. , & Levine, J. M. (2016). How variation between individuals affects species coexistence. Ecology Letters, 22, 19–33. 10.1111/ele.12618 27250037

[ece39254-bib-0025] He, N. P. , Liu, C. C. , Tian, M. , Li, M. L. , Yang, H. , Yu, G. R. , Guo, D. L. , Smith, M. D. , Yu, Q. , & Hou, J. H. (2018). Variation in leaf anatomical traits from tropical to cold‐temperate forests and linkage to ecosystem functions. Functional Ecology, 32, 10–19.

[ece39254-bib-0026] Hector, A. , Hautier, Y. , Saner, P. , Wacker, L. , Bagchi, R. , Joshi, J. , Scherer‐Lorenzen, M. , Spehn, E. M. , Bazeley‐White, E. , Weilenmann, M. , Caldeira, M. C. , Dimitrakopoulos, P. G. , Finn, J. A. , Huss‐Danell, K. , Jumpponen, A. , Mulder, C. P. H. , Palmborg, C. , Pereira, J. S. , Siamantziouras, A. S. D. , … Loreau, M. (2010). General stabilizing effects of plant diversity on grassland productivity through population asynchrony and overyielding. Ecology, 91, 2213–2220. 20836442 10.1890/09-1162.1

[ece39254-bib-0027] Isbell, F. I. , Polley, H. W. , & Wilsey, B. J. (2009). Biodiversity, productivity and the temporal stability of productivity: Patterns and processes. Ecology Letters, 12, 443–451. 19379138 10.1111/j.1461-0248.2009.01299.x

[ece39254-bib-0028] Ives, A. R. (1995). Predicting the response of populations to environmental change. Ecology, 75, 926–941.

[ece39254-bib-0029] Ives, A. R. , & Carpenter, S. R. (2007). Stability and diversity of ecosystems. Science, 317, 58–62. 17615333 10.1126/science.1133258

[ece39254-bib-0030] Lehman, C. L. , & Tilman, D. (2000). Biodiversity, stability, and productivity in competitive communities. American Naturalist, 156, 534–552. 10.1086/303402 29587515

[ece39254-bib-0031] Levine, J. M. , Bascompte, J. , Adler, P. B. , & Allesina, S. (2017). Beyond pairwise mechanisms of species coexistence in complex communities. Nature, 546, 56–64. 28569813 10.1038/nature22898

[ece39254-bib-0032] Li, F. , Zhou, G. Y. , Yang, L. C. , Xu, W. H. , Zhong, Z. B. , & Song, W. Z. (2013). Effect of fence on biodiversity and stability of the main plant communities in the Qinghai Lake area. Research of Soil and Water Conservation, 20, 135–140.

[ece39254-bib-0034] Liu, N. , Yu, L. F. , Zhao, Q. , Wu, Y. N. , & Yan, L. B. (2020). C:N:P stoichiometry of leaf‐litter‐soil continuum in secondary forests of the rocky desertification regions of the karst plateau. Chinese Journal of Applied & Environmental Biology, 26, 681–688.

[ece39254-bib-0035] Liu, W. W. , Yu, L. F. , Yan, L. B. , Liu, N. , & Zhao, Q. (2019). Analysis of soil fungal community composition at different stages of vegetation restoration in karst stone desertification areas. Journal of Ecology and Environment, 28, 669–675.

[ece39254-bib-0036] Loreau, M. , & de Mazancourt, C. (2013). Biodiversity and ecosystem stability: A synthesis of underlying mechanisms. Ecology Letters, 16, 106–115. 23346947 10.1111/ele.12073

[ece39254-bib-0037] Lv, G. H. , Du, X. , Yang, J. J. , Ma, Y. , & Meng, J. X. (2007). Community stability of deserts vegetation at Fukang oasis‐desert ecotone. Arid Land Geography, 5, 660–665.

[ece39254-bib-0038] Macarthur, R. (1955). Fluctuations of animal populations and a measure of community stability. Ecology, 36, 533–536.

[ece39254-bib-0039] Maestre, F. T. , Quero, J. L. , & Gotelli, N. J. (2012). Plant species richness and ecosystem multifunctionality in global drylands. Science, 335, 214–218. 22246775 10.1126/science.1215442 PMC3558739

[ece39254-bib-0040] May, R. M. (1973). Stability and complexity in model ecosystems. Monographs in Population Biology, 6, 1–235. 4723571

[ece39254-bib-0041] Mccann, K. S. (2000). The diversity‐stability debate. Nature, 405, 228–233. 10821283 10.1038/35012234

[ece39254-bib-0042] Mori, A. S. , Furukawa, T. , & Sasaki, T. (2013). Response diversity determines the resilience of ecosystems to environmental change. Biological Reviews, 88, 349–364. 23217173 10.1111/brv.12004

[ece39254-bib-0043] Mougi, A. , & Kondon, M. (2012). Diversity of interaction types and ecological community stability. Science, 337, 349–351. 22822151 10.1126/science.1220529

[ece39254-bib-0044] Mueller‐Dombois, D. , & Ellenberg, H. (1974). Aims and methods of vegetation ecology (p. 547). John Wiley and Sons.

[ece39254-bib-0045] Naeem, S. , & Li, S. B. (1997). Biodiversity enhances ecosystem reliability. Nature, 390, 507–509.

[ece39254-bib-0046] Niu, K. C. , Choler, P. , de Bello, F. , Mirotchnick, N. , Du, G. Z. , & Sun, S. C. (2014). Fertilization decreases species diversity but increases functional diversity: A three‐year experiment in a Tibetan alpine meadow. Agriculture, Ecosysytems & Enviroment, 182, 106–112.

[ece39254-bib-0047] Odum, E. P. (1953). Fundamentals of ecology. Saunders.

[ece39254-bib-0048] Pérezharguindeguy, N. , Díaz, S. , & Garnier, E. (2013). New handbook for standardised measurement of plant functional traits worldwide. Australian Journal of Botany, 61, 167–234.

[ece39254-bib-0049] Petchey, O. L. , Evans, K. L. , Fishburn, I. S. , & Gaston, K. J. (2007). Low functional diversity and no redundancy in British avian assemblages. Journal of Animal Ecology, 76, 977–985. 17714276 10.1111/j.1365-2656.2007.01271.x

[ece39254-bib-0050] Pillar, V. D. , Blanco, C. C. , Müller, S. C. , Sosinski, E. E. , Joner, F. , & Duarte, L. D. S. (2013). Functional redundancy and stability in plant communities. Journal of Vegetation Science, 24, 963–974.

[ece39254-bib-0051] Pocock, M. J. O. , Evans, D. M. , & Memmott, J. (2012). The robustness and restoration of a network of ecological networks. Science, 335, 973–977. 22363009 10.1126/science.1214915

[ece39254-bib-0052] Polley, H. W. , Wilsey, B. J. , & Derner, J. D. (2007). Dominant species constrain effects of species diversity on temporal variability in biomass production of tallgrass prairie. Oikos, 116, 2044–2052.

[ece39254-bib-0053] R Core Team . (2020). R: A language and enviroment for statistical computing. R Foundation for Statistical Computing.

[ece39254-bib-0054] Rao, C. R. (1982). Diversity and dissimilarity coefficients: A unified approach. Theoretical Population Biology, 21, 24–43.

[ece39254-bib-0055] Ren, H. , Shen, W. , Lu, H. , Wen, X. , & Jian, S. (2007). Degraded ecosystems in China: Status, causes, and restoration efforts. Landscape and Ecological Engineering, 3, 1–13.

[ece39254-bib-0056] Ricotta, C. , Bacaro, G. , Caccianiga, M. , Cerabolini, B. E. L. , & Pavoine, S. (2018). A new method for quantifying the phylogenetic redundancy of biological communities. Oecologia, 186, 339–346. 29209844 10.1007/s00442-017-4026-x

[ece39254-bib-0057] Ricotta, C. , de Bello, F. , Moretti, M. , Caccianiga, M. , Cerabolini, B. E. L. , & Pavoine, S. (2016). Measuring the functional redundancy of biological communities: A quantitative guide. Methods in Ecology and Evolution, 7, 1386–1395.

[ece39254-bib-0058] Ricotta, C. , Laroche, F. , Szeidl, L. , & Pavoine, S. (2020). From alpha to beta functional and phylogenetic redundancy. Methods in Ecology and Evolution, 11, 487–493.

[ece39254-bib-0059] Rosenfeld, J. S. (2002). Functional redundancy in ecology and conservation. Oikos, 98, 156–162.

[ece39254-bib-0060] Sasaki, T. , & Lauenroth, W. K. (2011). Dominant species, rather than diversity, regulates temporal stability of plant communities. Oecologia, 166, 761–768. 21279386 10.1007/s00442-011-1916-1

[ece39254-bib-0061] Sasaki, T. , Okubo, S. , Okayasu, T. , Jamsran, U. , Ohkuro, T. , & Takeuchi, K. (2009). Two‐phase functional redundancy in plant communities along a grazing gradient in Mongolian rangelands. Ecology, 90, 2598–2608. 19769137 10.1890/08-1850.1

[ece39254-bib-0062] Simpson, E. (1949). Measurement of diversity. Nature, 163, 688.

[ece39254-bib-0063] Sitzia, T. , Campagnaro, T. , & Kowarik, I. (2015). Using forest management to control invasive alien species: Helping implement the new European regulation on invasive alien species. Biological Invasions, 18, 1–7.

[ece39254-bib-0064] Song, T. Q. , Wang, K. L. , Zeng, F. P. , Peng, W. X. , & Du, H. U. (2015). Plant and the environment in karst areas of Southwest China. Science Press.

[ece39254-bib-0065] Taylor, L. R. (1961). Aggregation variance and the mean. Nature, 189, 732–735.

[ece39254-bib-0066] Tilman, D. (1994). Competition and biodiversity in spatially structured habitats. Ecology, 75, 2–16.

[ece39254-bib-0067] Tilman, D. (1996). Biodiversity: Population versus ecosystem stability. Ecology, 77, 350–363.

[ece39254-bib-0068] Tilman, D. (1999). The ecological consequences of changes in biodiversity: A search for general principles. Ecology, 80, 1455–1474.

[ece39254-bib-0070] Tilman, D. , Reich, P. B. , & Knops, J. M. (2006). Biodiversity and ecosystem stability in a decade‐long grassland experiment. Nature, 441, 629–632. 16738658 10.1038/nature04742

[ece39254-bib-0071] Valone, T. J. , & Hoffman, C. D. (2003). A mechanistic examination of diversity‐stability relationships in annual plant communities. Oikos, 103, 519–527.

[ece39254-bib-0072] Walker, B. H. (1992). Biological diversity and ecological redundancy. Conservation Biology, 6, 18–23.

[ece39254-bib-0073] Walker, B. H. (1995). Conserving biological diversity through ecosystem eesilience. Conservation Biology, 9, 747–752.

[ece39254-bib-0074] Wang, H. F. , Lv, G. H. , Zhou, Y. Z. , & Cao, J. (2017). Effects of functional diversity and functional redundancy on the stability of desert plant communities under different water and salt gradients. Acta Ecologica Sinica, 37, 7928–7937.

[ece39254-bib-0075] Wang, S. J. (2003). The most serious eco‐geologically environmental problem in southwest rocky desertification. Bulletin of Mineral, Petrology Geochemistry, 23, 120–126.

[ece39254-bib-0076] Wei, T. Y. , & Viliam S . (2021). R package 'corrplot': Visualization of a correlation matrix (Version 0.92). https://github.com/taiyun/corrplot

[ece39254-bib-0077] Wickham, H. (2016). ggpplot2: Elegant graphics for data analysis. Springer‐Verlag.

[ece39254-bib-0078] Wood, C. M. , Mckinney, S. T. , & Loftin, C. S. (2017). Intraspecific functional diversity of common species enhances community stability. Ecology and Evolution, 7, 1553–1560. 28261464 10.1002/ece3.2721 PMC5330891

[ece39254-bib-0079] Yao, T. H. , Zhu, Z. H. , Li, Y. N. , Pan, S. Y. , Kong, B. B. , Wei, X. H. , & Du, J. L. (2016). Effects of functional diversity and functional redundancy on the community stability of an alpine meadow. Acta Ecologica Sinica, 36, 1547–1558.

[ece39254-bib-0080] Yu, L. F. , Zhu, S. Q. , Wei, L. M. , Chen, Z. R. , & Xiong, C. B. (1998). Study on the natural restoration process of degraded karst communities—Successional sere journal of mountain agriculture and biology. Journal of Mountain Agricultural Biology, 17, 71–77.

[ece39254-bib-0081] Yu, L. F. , Zhu, S. Q. , Ye, J. Z. , Wei, L. M. , & Chen, Z. R. (2000). A study on evaluation of natural restoration for degraded karst forest. Scientia Silvae Sinicae, 46, 12–19.

[ece39254-bib-0082] Yu, L. F. , Zhu, S. Q. , Ye, J. Z. , Wei, L. M. , & Chen, Z. R. (2002). Dynamics of a degraded karst forest in the process of natural restoration. Scientia Silvae Sinicae, 48, 1–7.

